# Comment: In Response to “Auer Rod-Like Inclusions in Reactive Plasma Cells in a Case of Acute Myeloid Leukemia”

**DOI:** 10.4274/tjh.2016.0139

**Published:** 2016-12-01

**Authors:** Smeeta Gajendra

**Affiliations:** 1 Medanta-The Medicity, Department of Pathology and Laboratory Medicine, Gurgaon, India

**Keywords:** Plasma cell, Inclusion, Reactive plasmacytosis

## To the Editor,

I read the article “Auer Rod-Like Inclusions in Reactive Plasma Cells in a Case of Acute Myeloid Leukemia” by Pradhan when it was first published online (http://www.journalagent.com/tjh/pdfs/TJH-09216-IMAGES_IN_HEMATOLOGY-PRADHAN.pdf). The manuscript is well written with the description of a rare presence of Auer rod-like inclusions in reactive plasma cells in a case of acute myeloid leukemia (AML). However, it is not the first case of Auer rod-like inclusions in reactive plasma cells in a case of AML in the literature as was claimed by the author in the article. Sharma et al. had already described a case of the presence of this type of plasma cell inclusion in a case of therapy-related AML. Needle-like or Auer rod-like intracytoplasmic inclusions in plasma cells were first described by Steinmann in 1940. A few cases of multiple myeloma with intracytoplasmic plasma cell inclusions are described in the literature [1]. Other conditions associated with these crystalline intracytoplasmic inclusions are plasmacytoma, chronic lymphocytic leukemia, lymphoplasmacytic lymphoma, mucosa-associated lymphoid tissue lymphomas, and, rarely, high-grade lymphomas [2]. Lemez reported a very rare case of Auer rod-like inclusions in reactive plasma cells in a patient with aplastic anemia [3]. Some postulations were described in the literature that these inclusions are related to abnormal synthesis, trafficking, or excretion of the immunoglobulin or immunoglobulin light chains that accumulate in excess within the cytoplasm [4], but immunocytochemical examinations revealed no reaction with antibodies against immunoglobulins, light chains, or amyloid A antibodies inside the inclusions [5]. These are positive for α-naphthyl acetate esterase (sensitive to sodium fluoride treatment) and β-glucuronidase, suggesting a lysosomal origin [1]. Plasmacytosis in AML occurs in about 7% of cases and the number of plasma cells may vary from 5% to 16%. This plasmacytosis is due to increased production of IL-6 by leukemic blasts, causing stimulation of plasma cells resulting in marrow plasmacytosis [6]. A rare case of Auer rod-like inclusions in reactive plasma cells in a case of AML was reported by Sharma et al. [7]. This should be diagnosed with caution to exclude the coexistence of multiple myeloma with AML. Not only serum and urine protein electrophoresis with immunofixation but also serum free light-chain assay should be performed to exclude associated nonsecretory myeloma.

Conflict of Interest: The author of this paper has no conflicts of interest, including specific financial interests, relationships, and/or affiliations relevant to the subject matter or materials included.
